# Health Consequences of the COVID-19 Pandemic among Health-Care Workers: A Comparison between Groups Involved and Not Involved in COVID-19 Care

**DOI:** 10.3390/healthcare10122540

**Published:** 2022-12-15

**Authors:** Malin Lohela-Karlsson, Emelie Condén Mellgren

**Affiliations:** 1 Department of Public Health and Caring Sciences—Health Services Research, Uppsala University, P.O. Box 564, SE-751 22 Uppsala, Sweden; 2 Centre for Clinical Research, Region Västmanland—Uppsala University, Hospital of Västmanland, SE-721 89 Vasteras, Sweden; 3 Centre for Musculoskeletal Research, Department of Occupational Health Sciences and Psychology, University of Gävle, SE-801 76 Gävle, Sweden

**Keywords:** health care, COVID-19, mental health, occupational health, health care workers

## Abstract

Health consequences have been reported among health-care workers (HCWs) exposed to COVID-19. Sweden chose to manage the pandemic with a lower and more equal long-lasting work strain and shorter periods of recovery than in other countries. Few studies have examined the health consequences among HCWs working in such conditions. This study compared the health consequences after the first wave of the COVID-19 pandemic between HCWs involved in the care of COVID-19 patients and other HCWs and between occupational groups working in COVID-19 care. Multinomial logistic regression and univariate general linear models were used to identify differences. The levels of depression, emotional and physical fatigue, sleep quality, and general health were measured 6 months after the onset of the pandemic in 3495 HCW employed in Sweden. HCWs directly involved in COVID-19 care reported significantly poorer sleep quality and higher scores on emotional and physical exhaustion than those not involved in such care. Health consequences did not differ significantly between different occupational groups involved in COVID-19 care except for specialist nurses/midwives. HCWs more frequently involved in COVID-19 care reported higher levels of emotional and physical fatigue and poorer sleep but less severe than those reported in more severely affected countries.

## 1. Introduction

During the COVID-19 pandemic, the health-care system saw extensive changes, which had a major impact on the work situation of health-care workers (HCWs). In the Swedish health-care system, employees were urged to collaborate to solve problems that none had previously encountered. The number of intensive care units quadrupled, and extensive effort was needed to secure sufficient protective equipment. Inpatient care places were reorganized, and new routines were introduced to reduce the risk of infection in hospital care, as well as in home care [1]. Care was provided in different ways, for example, greater use of digital interactions, more frequent care at home, and the use of tents and barracks to separate care flows. Infection tracing continued but with a different focus throughout the pandemic. Nearly all HCW were affected by the pandemic according to their assigned tasks within the health-care system. For example, several HCW were redeployed from their usual workplace to higher-risk front-line jobs when the usual practices were disrupted [2]. The COVID-19 pandemic challenged the health-care system as well as the work environment for all HCWs and the system’s ability to maintain quality of care. Besides affecting work organizations and routines, the psychosocial work environment was negatively affected for HCW working with COVID-19 patients [3,4,5] with associated health consequences [6]. Studies of the short-term health consequences for HCWs related to the pandemic’s outbreak in different countries have reported a considerable impact on the psychological well-being of frontline HCWs [7,8,9,10]. Nurses and employees in close contact with COVID-19 patients have been shown to have a higher risk of adverse mental health outcomes than other workers [7,8,9], and long-term consequences are expected. A more recent study indicate that health consequences also are related to individual conditions, such as age and work experience [11]. Internationally, previous outbreaks of severe acute respiratory syndrome (SARS) increased the risk of long-term mental health consequences, as shown by higher levels of burnout, psychological distress, and posttraumatic stress, in HCWs directly involved with SARS patients compared with other employees [12].

These earlier findings suggest that, during the COVID-19 pandemic, HCWs may have had a higher risk of similar adverse long-term mental health problems. In addition, reduced performance, poor quality of care [13], increased sickness absence [14], and turnover intentions could be expected [15]. The COVID-19 pandemic differs from previous outbreaks because it has affected almost all HCWs to some extent, and it is possible that the health of other health-related occupational groups has also been affected. However, there is limited knowledge about how various occupational groups and different groups of HCWs have been affected by the pandemic. For example, little is known about the consequences of COVID-19 for the primary care workforce [7] and in different settings, and the structural strategies [16,17].

Unlike many other countries, Sweden chose a different approach to managing the COVID-19 pandemic [1,18]. Using a public health strategy to slow the spread of the virus, Sweden sought to flatten the epidemic curve, which is described as a visual depiction of the number of infected people needing health care over time. Flattening the curve implies slowing the spread of the epidemic to reduce the peak number of people requiring care at any given time to ensure that demand does not exceed the capacity of the health-care system [19]. Such a strategy relies on mitigation techniques such as handwashing, use of face masks, and social distancing. Sweden did not officially lock down, although Swedes were recommended to observe social distancing in public, refrain from nonessential travel, and work from home when possible. The pandemic exposed Swedish HCWs to a new situation never before experienced. The Swedish approach required lower and more equal long-lasting work strain and shorter periods of recovery compared with other countries. Little is known about the consequences of this type of strategy on HCWs.

Previous studies have examined frontline HCWs only, and few have compared health consequences between various groups of HCWs during the same period. Most previous studies have included employees from specific occupational HCW groups within the same organization or from several organizations around the country. Given that the pandemic has affected the work situation for almost all occupational groups, although some differently depending on the work tasks and workplace, it is possible that these consequences have impacted groups of HCWs differently. Workers who have been more exposed to the pandemic, for example, HCWs involved directly in the care of COVID-19 patients, may have experienced worse health consequences than other groups of HCWs. Knowledge about the potential health consequences among frontline HCWs in different occupational groups is needed to understand how the pandemic has affected different groups working in the same work environment.

The overall aim of the present study was to examine health consequences among HCWs after the first wave of the COVID-19 pandemic. We hypothesized that HCWs involved in the care of COVID-19 would report more health consequences compared with other HCWs. We also hypothesized that the health consequences would differ between occupational groups working in COVID-19 care.

## 2. Materials and Methods

### 2.1. Data Collection

A cross-sectional survey of the work environment and patient safety culture was conducted as part of the yearly employee survey in a Swedish region in October 2020. Regions in Sweden are responsible for tasks that are common to large geographical areas and that often require large financial resources. This includes, for example, health-care services, the public transport system, and culture, and ways to strengthen the growth and development of the regions.

All employees who had been working at least 40% of a full-time equivalent for 5 months or longer were invited to complete the questionnaire. The invitation was sent by email and followed up with three reminders. Responses were anonymous to the employer and were collected and summarized by an external consultant. Employees were encouraged to participate, but participation was voluntary. Data were collected and reported at the group level. The questionnaire used to collect the data included validated questions on psychosocial work environment factors, health conditions, productivity, and patient safety culture, as well as background information, such as age, gender, managerial position, and occupation.

### 2.2. Setting and Study Population

On 9 March 2020, the first COVID-19-positive patient was detected in the study region. This was followed by a rapidly increasing incidence of illness and many patients who required inpatient and intensive care. The peak of the first wave relating to inpatient need occurred in the middle of April 2020. The questionnaire was sent to all employees (n = 5594, response rate 77%) in the health-care service in the region about 6 months after the first confirmed COVID-19 case in the study region. The health-care service is divided into primary care and specialist care. HCWs in primary care services include general medical practitioners, specialist nurses, physiotherapists, and occupational therapists who provide care to people without severe symptoms. Specialist care provides care for those requiring more specialized medical measures than what is available through primary care. Only HCW were included in the study population. Employees employed in other type of occupations, for example administrators, medical secretaries, psychologists, engineers or similar, were excluded. Both HCWs from primary care and specialist care were included, and all were involved to some extent in the care of patients with suspected or confirmed COVID-19 infection. 

### 2.3. Measurements

#### 2.3.1. Background Characteristics

Age, gender, managerial position, occupation, and workplace were included as background characteristics. Occupation was divided into following categories group/team leader, physician, consultant/general practitioner, resident intern, nurse, specialist nurse or midwife, medical laboratory, nurse assistant, and rehabilitation therapist.

#### 2.3.2. Explanatory Variables

All HCWs employed in health care were asked a question about whether they had been involved in COVID-19 treatment or care in the past 6 months. COVID-19 treatment or care was defined as participation in the care of COVID-19 patients, for example, through work in a ward, consultancy, out-patient clinic or laboratory, or participation in patient examination, treatment, transport, or telephone or other forms of counseling. The response options were “yes, daily”, “yes, weekly”, “yes, monthly,” “yes, a few times”, and “no”. Those answering “yes, daily” or “yes, weekly” were categorized as having participated in COVID-19 care. Those answering “yes, monthly” or “yes, a few times” were categorized as frequently participating in COVID-19 care a few times. Employees responding “no” were included in the group not involved in COVID-19 care. 

#### 2.3.3. Outcome Variables

Health outcomes were measured using different measures. Sleep quality was measured using four questions from the sleep quality index in the validated Karolinska Sleep Questionnaire [20]. Each item had a six-point response option ranging from “never” (0 points) to “always (5 times per week or more)” (5 points). The total score was calculated from the mean scores of all items (range 0–5) and a higher value indicated poorer sleep quality.

General health was measured using a validated question from World health organization general health (WHO-GH) questionnaire [21], which has five response option ranging from “poor” to “excellent.” The response options were categorized into three groups “poor/fair”, “good”, and “very good/excellent”. 

Emotional and physical fatigue was measured using the emotional and physical exhaustion (EPE) subscale of the validated Shirom–Melamed Burnout Questionnaire (SMBQ) [22,23], which comprises eight items. Each item is rated using a seven-point scale (1 = “almost never” to 7 = “almost always”). The total score was calculated from the mean scores of all items (range 1–7) and a higher value indicated more severe problems. 

Depression was measured using the Hospital Anxiety Disorder Depression scale (HAD-D). This scale includes seven questions, each with four response options ranging from “not at all” to “nearly all the time” and are summed to generate a score from 0 to 21. The cutoff scores are used to indicate non-depression (0–7.99), low risk of depression (8.00–11.00), and high risk of depression (11.01–21.00) [24].

### 2.4. Statistical Analysis

The health consequences of the COVID-19 pandemic among HCWs were analyzed using different methods. To examine the first aim, multinominal logistic regressions were used to analyze the associations between working in COVID-19 care and depression as well as general health among HCWs. The analyses were controlled for age, gender, and occupation. The univariate general linear model (GLM) was used to analyze the associations between working in COVID-19 care and emotional and physical fatigue (EPE score) as well as sleep quality among HCWs. The analyses were controlled for age, gender, and occupation.

To examine the second aim, whether the health consequences differed between occupational groups working in COVID-19 care, only HCWs who had been involved in COVID-19 care daily or weekly were included in the analyses; managers were excluded. Occupational group was used as an explanatory variable, and age and gender were included as confounders. Multinominal logistic regression was conducted to examine associations with depression and general health. The univariate GLM was used to examine associations with emotional and physical fatigue as well as sleep quality. All statistical analyses were performed using IBM SPSS Statistics (version 28).

## 3. Results

### 3.1. Descriptive Statistics

The descriptive statistics of the study sample are shown in Table 1 (n = 3495). About 39% of the sample had been involved in COVID-19 care more than once weekly during the first 6 months of the pandemic, 24% had been involved a few times, and 37% had not been involved in any COVID-19 care during this period.

### 3.2. Health Consequences of the COVID-19 Pandemic among HCWs

HCWs who were involved in COVID-19 care on a weekly basis or more often had a higher odds ratio (1.810, 95% confidence interval (CI) 1.115–2.937) of experiencing a high risk of depression compared with those not involved in COVID-19 care (Table 2). However, the odds ratio was no longer significant after adjustment for the confounders (1.490, 95% CI 0.888–2.500). This result indicates that HCWs involved in COVID-19 care did not have an increased risk of depression compared with those not involved in COVID-19 care. The odds ratio for experiencing impaired general health was lower among HCWs involved in COVID-19 care than in those not involved in COVID-19 care (Table 2).

HCWs who worked in COVID-19 care reported significantly poorer sleep quality than those not involved in COVID-19 care (Table 3). This association remained significant after adjustment for confounders (Involved few times β = 0.133 and Involved weekly β = 0.135, respectively). HCWs involved in COVID-19 care also reported significantly higher levels of emotional and physical fatigue than those who did not work in COVID-19 care. This association remained significant after adjustment for confounders (involved few times β = 0.141 and involved weekly β = 0.317, respectively).

### 3.3. Differences in Health Consequences between Occupational Groups Working in COVID-19 Care

Descriptive data for the HCWs involved in COVID-19 care are presented for the different occupational groups in Table 4. The occupational groups consultant/general practitioners and resident intern contains of a larger proportion of employees that report better general health and no risk of depression than in other occupational groups. The mean score of sleep quality as well as mental and physical fatigue are also better in these two occupational groups.

The odds ratios of experiencing impaired general health were higher in the medical laboratory group (3.538, 95% CI, 1.166–10.740), nurses (1.853, 95% CI, 1.063–3.228), and specialist nurses/midwives (2.312, 95% CI, 1.258–4.249) than in physicians. The increased risk was no longer significant for the medical laboratory group and nurses after adjusting for age and gender. No other significant health consequences differed between the occupational groups and physicians (Table 5 and Table 6).

## 4. Discussion

The overall aim of the present study was to identify the health consequences of the COVID-19 pandemic among HCWs. It was hypothesized that HCWs involved in COVID-19 care would report more health consequences than other HCWs and that the health consequences would differ between occupational groups involved in COVID-19 care. HCWs involved in COVID-19 care reported poorer sleep quality and higher scores on the EPE subscale than those not involved in such care. However, despite the higher levels of exhaustion and poorer sleep quality in HCWs involved in COVID-19 care, their general health was significantly better than those not involved in such care. No significant differences in health consequences were found between occupational groups involved in COVID-19 care, except for the occupational group specialist nurses/midwives who had a higher odds ratio of impaired general health compared with physicians.

Short-term health consequences for HCWs close to the outbreak in different countries have been reported in several studies. These studies have found a considerable impact on psychological well-being on frontline HCWs, especially in nurses and other employees in close contact with COVID-19 patients who were at higher risk of adverse mental health outcomes than others [7,8]. The data used in the present study were collected about 6 months after the first wave of the pandemic hit Sweden, a period that could be considered as short term. As in other published studies, the present study also found health consequences among HCWs that had been involved in COVID-19 care. This study adds to information about differences in health consequences between groups of HCWs within the same organization. For example, HCWs involved in COVID-19 care reported higher levels of emotional and physical fatigue compared with those not involved in such care. The more exposed HCWs were, in terms of time, the higher the reported levels. 

Normative values have been reported for the public sector and the EPE subscale of the SMBQ [25]. Slightly higher values were found in this population than the normative values, which may be a consequence of the pandemic, and the higher values for HCWs more frequently involved in COVID-19 care indicate support to such hypothesis. In the current study, the EPE score ranged from exceptionally low to exceptionally high, with an average value of 4.00, which is considered to be moderately high (75th percentile of the population) [25]. Among those involved in COVID-19 care, a larger proportion of the HCWs than in the general population had EPE scores that are considered moderately high, which indicates symptoms of exhaustion and may have consequences for patient safety and quality of care. However, compared with the results from other countries, a smaller proportion of the HCWs in this study population reported adverse mental health problems after the first wave of the pandemic [26,27]. 

HCWs involved in COVID-19 care reported significantly poorer sleep quality than other HCWs in the organization. Despite the statistically significant difference, this may not be a clinically relevant difference. In a previous study, normative data for poor sleep quality for the general population was reported as 1.58 on a scale of 0–5, where higher values indicate poorer sleep quality [20]. In the current study, HCWs not involved in COVID-19 care reported an average sleep quality score of 1.96, which is higher than in the general population. The other groups had even higher average scores, which suggests that this study population had poorer sleep quality than should be expected under normal circumstances. The 90th percentile is sometimes used as a cutoff to indicate sleep problems. A larger proportion in the present study reported sleep problems compared with the general population: 90th percentile score of 3.5 versus 3.0, respectively [20]. This difference could be interpreted as indicating that a larger proportion of the employees in this study population have problems with sleep quality than what is common in the general population.

A previous study found that people with high scores for mental and physical exhaustion also report sleep problems [28]. Similar results were found in the present study in which both poorer sleep quality and higher scores on emotional and physical exhaustion were more frequent among those involved in COVID-19 care. Despite the reporting of some symptoms of poor health in HCWs involved in COVID-19 care, their perceived general health was better than those who had not been involved in COVID-19 care. The reason for this difference should be explored further. In contrast to previous studies [7,9], we found no differences in mental health problems between occupational groups in the present study. 

Overall, the results of this study suggest that being exposed to COVID-19 care in general seems to be more relevant to the evaluated health consequences than the work task of HCWs in the current situation. However, it is possible that the differences do not only relate to occupational groups in general, independent of the frequency of and perceived stress associated with being exposed to COVID-19. Instead, differences may be related to a combination of occupational groups in different parts of a health-care organization with work tasks related to COVID-19 care, for example, the combination of frequency and severity of exposure. Our hypothesis that HCWs more frequently involved in the care of COVID-19 patients would report more health consequences compared with other HCWs was supported by our findings, but not to the extent expected, especially compared with reports from other more severely affected countries [29]. One possible explanation is that, by flattening the curve, the Swedish model slowed the spread of the epidemic, which reduced the peak number of people requiring care at a given time and prevented demand for health care from exceeding the system’s capacity. This may have avoided the most severe scenarios experienced by HCWs in other countries. Another explanation could be related to resilient capacity of the organization. Comparison to health consequences of HCW in other health care organizations in Sweden are needed to investigate if the present study population differ from other similar populations.

A strength of this study is that it included HCWs from the same organization who had been exposed to COVID-19 during the same period, which made it possible to compare health consequences between those involved and not involved in COVID-19 care. This has seldom been done in other studies of the health consequences for HCWs in specific health-care settings. Another strength of this study is the opportunity to compare health consequences between different types of occupational groups among HCWs. This information adds to the knowledge of whether health consequences among HCWs are related to COVID-19 itself or to the types of work tasks and work situations related to such care. However, there are also limitations with the study. The data collection, which took place during a limited period of time (three weeks) and sent out to employees work e-mails, poses a risk for a selected population as the employees with the greatest vulnerability hypothetical were absent due to sickness.

In the present study, a self-rated and broadly defined questionnaire was used to report whether HCW had been involved in COVID-19 care and the frequency of this involvement. This approach may lead to a mix of HCWs involved in different types of COVID-19 care, which in turn may influence how they were affected by it. Future studies should examine the combination of the severity of exposure to COVID-19 with the frequency of exposure both in general, as well as for different types of occupational groups, to understand better how HCWs are affected by extreme and unknown situations at work. The long-term consequences of such exposure with limited possibility for recovery, as in the Swedish case, with a lower and more equal long-lasting work strain should also be investigated.

## 5. Conclusions

The overall findings of this study suggest that 6 months after the outbreak of the COVID-19 pandemic in Sweden, HCWs involved in COVID-19 care reported poorer sleep quality and higher levels of emotional and physical fatigue than normative data for the general Swedish population. The sleep quality and level of emotional and physical fatigue worsened with increasing frequency of involvement in COVID-19 care. However, the health consequences in the current population were not as severe as those found among HCWs in more severely affected countries. These results may suggest clinical implications in terms of preventive interventions targeting the psychosocial work environment as well as possibility of interventions aiming for healthy sleep hygiene when conditions change significantly. However, the long-term effects of the lower, long-lasting work strain and the short periods of recovery on HCW health and well-being as a result of the Swedish strategy should be evaluated further. 

## Figures and Tables

**Table 1:** Descriptive data for the total study population and employees working in COVID-19 care.

	Total Population	Involved in COVID-19 Care	Involved a Few Times in COVID-19 Care	Not Involved in COVID-19 Care
N (%)	N = 3495	N= 1352 (38.7)	N = 850 (24.3)	N= 1293 (37.0)
Gender, N (%)				
Female	2901 (83.1)	1067 (79.0)	703 (82.9)	1131 (87.7)
Male	580 (16.6)	278 (20.6)	144 (17.0)	158 (12.2)
Other gender identity	8 (0.3)	6 (0.4)	1 (0.1)	1 (0.1)
Age, years; N (%)				
≤29	438 (12.6)	210 (15.6)	129 (15.2)	99 (7.7)
30–39	813 (23.3)	347 (25.7)	193 (22.8)	273 (21.2)
40–49	885 (25.4)	358 (26.5)	213 (25.1)	314 (24.2)
50–59	915 (26.2)	306 (22.7)	210 (24.8)	399 (31.0)
≥60	436 (12.5)	129 (9.6)	103 (12.1)	204 (15.8)
Managerial position, N (%)				
Yes	270 (7.8)	72 (5.4)	49 (5.8)	149 (11.7)
No	3174 (92.2)	1262 (94.6)	789 (94.2)	1123 (88.3)
Occupation, N (%)				
Group/team leader	52 (1.6)	17 (1.3)	9 (1.1)	26 (2.3)
Physician	245 (7.7)	80 (6.3)	57 (7.2)	108 (9.6)
Consultant/general practitioner	142 (4.5)	60 (4.8)	45 (5.7)	37 (3.3)
Resident Intern	51 (1.6)	21 (1.7)	22 (2.8)	8 (0.7)
Nurse	989 (31.1)	446 (35.3)	237 (30.0)	306 (27.2)
Nurse (specialist, midwife)	360 (11.3)	168 (13.3)	69 (8.7)	123 (11.0)
Medical laboratory	101 (3.2)	28 (2.2)	14 (1.8)	59 (5.3)
Nurse assistant	935 (29.5)	424 (33.6)	268 (34.0)	243 (21.6)
Rehabilitation therapist	299 (9.4)	18 (1.4)	68 (8.6)	213 (19.0)
General health, N (%)				
Excellent/very good	1620 (46.4)	651 (48.4)	393 (46.2)	576 (44.6)
Good	1304 (37.4)	492 (36.6)	316 (37.2)	496 (38.4)
Fairly/poor	564 (16.2)	203 (15.1)	141 (16.6)	220 (17.0)
Depression, N (%)				
No depression (0–7 points)	3081 (89.9)	1176 (88.2)	752 (89.9)	1152 (91.5)
Low risk of depression (8–10 points)	250 (7.3)	110 (8.2)	59 (7.0)	81 (6.4)
High risk of depression (11–21 points)	100 (2.9)	48 (3.6)	26 (3.1)	26 (2.1)
Sleep quality, mean (SD)	1.8 (1.2)	1.9 (1.2)	1.9 (1.2)	1.8 (1.2)
25th percentile	1.00	1.00	1.00	0.75
50th percentile	1.75	1.75	1.75	1.50
75th percentile	2.75	2.70	2.75	2.50
90th percentile	3.50	3.50	3.50	3.50
SMBQ emotional and physical fatigue, mean (SD)	3.2 (1.2)	3.4 (1.2)	3.2 (1.2)	2.9 (1.1)
25th percentile	2.13	2.25	2.13	2.00
50th percentile	2.88	3.13	2.88	2.63
75th percentile	4.00	4.25	4.00	3.75
90th percentile	5.00	5.25	5.00	4.75

SD, standard deviation; SMBQ, Shirom–Melamed Burnout Questionnaire.

**Table 2:** Associations between involvement in COVID-19 care and health consequences (general health and depression) among health-care workers (N = 3488).

Variable	Involvement in COVID-19 Care	Depression (Unadjusted) OR (95% CI)	Depression (Adjusted) ^c^ OR (95% CI)	Variable	General Health (Unadjusted) OR (95% CI)	General Health (Adjusted) ^c^ OR (95% CI)
High risk of depression ^a^	Involved in COVID-19 care	1.810 (1.115–2.937)	1.490 (0.888–2.500)	Fair/poor ^b^	0.816 (0.654–1.019)	0.785 (0.620–0.995)
	Involved a few times in COVID-19 care	1.533 (0.883–2.661)	1.282 (0.713–2.304)		0.939 (0.734–1.203)	0.900 (0.693–1.170)
	Not involved in COVID-19 care	0	0		0	0
Low risk of depression ^a^	Involved in COVID-19 care	1.331 (0.988–1.794)	1.229 (0.893–1.693)	Good ^b^	0.878 (0.742–1.038)	0.821 (0.684–0.985)
	Involved a few times in COVID-19 care	1.117 (0.789–1.581)	1.122 (0.782–1.611)		0.934 (0.772–1.130)	0.910 (0.742–1.117)
	Not involved in COVID-19 care	0	0		0	0

^a^ Reference category: no risk of depression, ^b^ Reference category: very good/excellent health, ^c^ Adjusted for age, gender, and occupation, OR = odds ratio; CI = confidence interval.

**Table 3:** Associations between involvement in COVID-19 care and health consequences (sleep quality and emotional and physical fatigue) among health-care workers (N = 3488).

Variable	Sleep Quality (Unadjusted) R^2^ = 0.002 β (95% CI)	Sleep Quality (Adjusted) ^a^ R^2^ = 0.012 β (95%CI)	Emotional and Physical Fatigue (Unadjusted) R^2^ = 0.021 β (95% CI)	Emotional and Physical Fatigue (Adjusted) ^a^ R^2^ = 0.048 β (95% CI)
Intercept	1.762 (1.697–1.828)	1.957 (1.730–2.184)	2.949 (2.884–3.015)	3.840 (3.616–4.064)
Involved in COVID-19 care	0.106 (0.014–0.197)	0.135 (0.035–0.235)	0.405 (0.314–0.497)	0.317 (0.218–0.415)
Involved a few times in COVID-19 care	0.117 (0.013–0.221)	0.133 (0.021–0.245)	0.223 (0.119–0.327)	0.141 (0.030–0.251)
Not involved in COVID-19 care	0	0	0	0

^a^ Adjusted for age, gender, and occupation, CI = confidence interval.

**Table 4:** Descriptive data for employee health in different occupational groups of health-care workers involved in COVID-19 care (N = 1352).

Variable	Physician N = 80	Consultant/General Practitioner N = 60	Resident Intern N = 21	Nurse N = 446	Nurse (Specialist, Midwife) N = 168	Medical Laboratory N = 28	Nurse Assistant N = 424	Rehabilitation Therapist N = 18
General health, N (%)								
Excellent/very good	46 (57.5)	40 (66.7)	13 (61.9)	201 (45.3)	72 (42.9)	9 (32.1)	210 (49.9)	9 (50.0)
Good	21 (26.3)	14 (23.3)	6 (28.6)	170 (38.3)	76 (45.2)	10 (35.7)	145 (34.4)	4 (22.2)
Fair/poor	13 (16.3)	6 (10.0)	2 (9.5)	73 (16.4)	20 (11.9)	9 (32.1)	66 (15.7)	5 (27.8)
Depression, N (%)								
No depression	68 (85.0)	53 (91.4)	19 (90.5)	390 (87.8)	151 (89.9)	18 (69.2)	362 (87.7)	16 (88.9)
Low risk of depression	7 (8.8)	2 (3.4)	1 (4.8)	35 (7.9)	14 (8.3)	4 (15.4)	40 (9.7)	1 (5.6)
High risk of depression	5 (6.3)	3 (5.2)	1 (4.8)	19 (4.3)	3 (1.8)	4 (15.4)	11 (2.7)	1 (5.6)
Sleep quality, mean (SD)	1.83 (1.06)	1.46 (0.93)	1.55 (0.97)	1.93 (1.24)	1.89 (1.22)	2.09 (1.15)	1.89 (1.26)	2.13 (1.24)
Mental and physical fatigue, mean (SD)	3.14 (1.27)	3.00 (1.21)	3.04 (0.98)	3.44 (1.24)	3.38 (1.21)	3.63 (1.4)	3.41 (1.30)	3.20 (1.20)

**Table 5:** Associations between health consequences (general health and depression) and occupation in health-care workers involved weekly or daily in COVID-19 care (N = 1352).

Variable	Occupation	Depression (Unadjusted) OR (95% CI)	Depression (Adjusted) ^c^ OR (95% CI)	Variable	General Health (Unadjusted) OR (95% CI)	General Health (Adjusted) ^c^ OR (95% CI)
High risk of depression ^a^	Rehabilitation therapist	0.850 (0.093–7.787)	0.522 (0.053–5.093)	Fair/poor ^b^	1.966 (0.561–6.893)	1.212 (0.336–4.374)
	Nurse assistant	0.413 (0.139–1.227)	0.335 (0.107–1.047)		1.112 (0.566–2.184)	0.829 (0.413–1.664)
	Medical laboratory	3.022 (0.735–12.425)	2.720 (0.637–11.626)		3.538 (1.166–10.740)	2.752 (0.893–8.479)
	Nurse (specialist, midwife)	0.270 (0.063–1.163)	0.242 (0.055–1.072)		0.983 (0.446–2.166)	0.747 (0.333–1.676)
	Nurse	0.663 (0.239–1.834)	0.462 (0.155–1.374)		1.285 (0.657–2.515)	0.912 (0.453–1.833)
	Resident intern	0.716 (0.079–6.502)	0.271 (0.028–2.637)		0.544 (0.109–2.726)	0.345 (0.066–1.793)
	Consultant/general practitioner	0.770 (0.179–3.367)	0.409 (0.090–1.864)		0.531 (0.185–1.526)	0.459 (0.153–1.326)
	Physician	0	0		0	0
Low risk of depression ^a^	Rehabilitation therapist	0.607 (0.070–5.291)	0.492 (0.055–4.398)	Good ^b^	0.974 (0.269–3.522)	0.721 (0.196–2.650)
	Nurse assistant	1.073 (0.462–2.496)	1.005 (0.422–2.394)		1.512 (0.866–2.642)	1.279 (0.723–2.264)
	Medical laboratory	2.159 (0.569–8.193)	2.135 (0.553–8.245)		2.434 (0.862–6.872)	2.114 (0.743–6.013)
	Nurse (specialist, midwife)	0.901 (0.348–2.332)	0.876 (0.332–2.313)		2.312 (1.258–4.249)	1.981 (1.066–3.684)
	Nurse	0.872 (0.372–2.043)	0.757 (0.313–1.830)		1.853 (1.063–3.228)	1.499 (0.846–2.654)
	Resident intern	0.511 (0.059–4.416)	0.308 (0.035–2.752)		1.011 (0.338–3.026)	0.727 (0.236–2.239)
	Consultant/general practitioner	0.367 (0.073–1.838)	0.260 (0.051–1.326)		0.767 (0.345–1.703)	0.663 (0.294–1.496)
	Physician	0	0		0	0

^a^ Reference category: no risk of depression; ^b^ Reference category: very good/excellent health; ^c^ Adjusted for age and gender.

**Table 6:** Associations between health consequences (sleep quality and emotional and physical fatigue) and occupation among health-care workers involved in COVID-19 care weekly or daily (N = 1352).

Variable	Sleep Quality (Unadjusted) R^2^ = 0.009 β (95% CI)	Sleep Quality (Adjusted) ^a^ R^2^ = 0.019 β (95% CI)	Emotional and Physical Fatigue (Unadjusted) R^2^ = 0.010 β (95% CI)	Emotional and Physical Fatigue (Adjusted) ^a^ R^2^ = 0.041 β (95% CI)
Intercept	1.831 (1.565 to 2.098)	2.327 (1.912 to 2.742)	3.144 (2.868 to 3.419)	4.071 (3.646 to 4.496)
Rehabilitation therapist	0.294 (–0.328 to 0.916)	0.141 (–0.487 to 0.768)	0.058 (–0.585 to 0.701)	–0.235 (–0.877 to 0.407)
Nurse assistant	0.055 (–0.236 to 0.346)	–0.054 (–0.350 to 0.242)	0.268 (–0.032 to 0.569)	0.124 (–0.179 to 0.426)
Medical laboratory	0.258 (–0.265 to 0.782)	0.152 (–0.373 to 0.676)	0.481 (–0.060 to 1.022)	0.366 (–0.170 to 0.903)
Nurse (specialist, midwife)	0.054 (–0.270 to 0.378)	–0.055 (–0.383 to 0.274)	0.231 (–0.103 to 0.566)	0.106 (–0.230 to 0.441)
Nurse	0.094 (–0.195 to 0.384)	–0.019 (–0.316 to 0.278)	0.293 (–0.006 to 0.592)	0.094 (–0.210 to 0.397)
Resident/intern	–0.284 (–0.868 to 0.301)	–0.351 (–0.947 to 0.245)	–0.102 (–0.706 to 0.502)	–0.508 (–1.118 to 0.101)
Consultant/general practitioner	–0.369 (–0.776 to 0.038)	–0.356 (–0.769 to 0.057)	–0.142 (–0.563 to 0.279)	–0.355 (–0.777 to 0.068)
Physician	0	0	0	0

^a^ Adjusted for age and gender.

## Data Availability

The data presented in this study are available on request from the corresponding author.
